# Lipid level alteration in human and cellular models of alpha synuclein mutations

**DOI:** 10.1038/s41531-022-00313-y

**Published:** 2022-04-25

**Authors:** Hila Avisar, Cristina Guardia-Laguarta, Matthew Surface, Nikos Papagiannakis, Matina Maniati, Roubina Antonellou, Dimitra Papadimitriou, Christos Koros, Aglaia Athanassiadou, Serge Przedborski, Boaz Lerner, Leonidas Stefanis, Estela Area-Gomez, Roy N. Alcalay

**Affiliations:** 1 Department of Industrial Engineering & Management, Ben-Gurion University of the Negev, Beer-Sheva, Israel; 2 Department of Neurology, Columbia University Irving Medical Center, New York, NY USA; 3 First Department of Neurology, National and Kapodistrian University of Athens Medical School, Athens, Greece; 4 Center of Clinical Research, Experimental Surgery and Translational Research, Athens, Greece; 5 Second Department of Neurology, National and Kapodistrian University of Athens Medical School, Athens, Greece; 6 Department of Neurology, Henry Dunant Hospital Center, Athens, Greece; 7 Department of Biology, University of Patras Medical School, Athens, Greece; 8 Department of Pathology & Cell Biology and of Neuroscience, Columbia University, New York, NY USA; 9 Neurological Institute, Tel Aviv Sourasky Medical Center, Tel Aviv, Israel

**Keywords:** Translational research, Parkinson's disease

## Abstract

Lipid profiles in biological fluids from patients with Parkinson’s disease (PD) are increasingly investigated in search of biomarkers. However, the lipid profiles in genetic PD remain to be determined, a gap of knowledge of particular interest in PD associated with mutant α-synuclein (*SNCA*), given the known relationship between this protein and lipids. The objective of this research is to identify serum lipid composition from *SNCA* A53T mutation carriers and to compare these alterations to those found in cells and transgenic mice carrying the same genetic mutation. We conducted an unbiased lipidomic analysis of 530 lipid species from 34 lipid classes in serum of 30 participants with *SNCA* mutation with and without PD and 30 healthy controls. The primary analysis was done between 22 PD patients with *SNCA*+ (*SNCA*+*/*PD+) and 30 controls using machine-learning algorithms and traditional statistics. We also analyzed the lipid composition of human clonal-cell lines and tissue from transgenic mice overexpressing the same *SNCA* mutation. We identified specific lipid classes that best discriminate between *SNCA*+/PD+ patients and healthy controls and found certain lipid species, mainly from the glycerophosphatidylcholine and triradylglycerol classes, that are most contributory to this discrimination. Most of these alterations were also present in human derived cells and transgenic mice carrying the same mutation. Our combination of lipidomic and machine learning analyses revealed alterations in glycerophosphatidylcholine and triradylglycerol in sera from PD patients as well as cells and tissues expressing mutant α-Syn. Further investigations are needed to establish the pathogenic significance of these α-Syn-associated lipid changes.

## Introduction

Unbiased omics combined with bioinformatics are increasingly recognized as powerful approaches to gain insights into not only neurodegenerative disorders like Parkinson’s disease (PD), but also in unraveling meaningful markers of their complex pathological processes^1,2^. Among these global molecular approaches, investigations of the entire lipidome or selected lipids in idiopathic PD (i.e., non-carriers of known mutations for PD) have shown that changes in lipid profiles could indeed be valuable predictors of both motor and non-motor symptoms of the disease^3–5^. Moreover, the association between PD and lysosomal lipid hydrolases, specifically glucocerebrosidase (*GBA*) and potentially others (e.g., *SMPD1*), further supports the importance of exploring the role of lipids as biomarkers in idiopathic PD^6–8^. However, while idiopathic PD represents >90% of all PD cases, roughly 10% result from genetic mutations in a group of genes^9,10^. Thus far, lipid profiles in body fluids from these rare instances have not been examined.

Of all genes linked to PD, mutations in *SNCA*, which encodes α-synuclein (α-Syn), would be of major interest. Indeed, α-Syn and cellular lipids entertain bidirectional interactions: the presence of lipid rafts in specific cellular membranes attract α-Syn to these subcellular locations^11^, and in turn, α-Syn can regulate lipid metabolism via its location at the mitochondria associated endoplasmic reticulum (ER) membranes or MAM^11^, transient lipid-raft domain in the ER involved in the regulation of multiple lipid enzymes. Moreover, it was reported that α-Syn aggregation, which is a hallmark of PD pathology, may depend on the concentration of different lipids in cells and lysosomal membranes^12,13^. Here, we hypothesized that a-Syn contributes to the regulation of lipid homeostasis via its effect on the modulation of MAM activities and that we will be able to observe these changes in patients’ serum. We had a unique opportunity to measure 530 lipids in the serum of 22 PD patients carrying a *SNCA* mutation and 30 healthy controls using a random forest (RF) algorithm^14,15^, a widely used and accurate machine learning (ML) classifier that has been shown to be efficient and beneficial in various clinical studies^16–23^.

## Results

### Lipid *classes* in *SNCA*+/PD+

The best classification measures by the RF ML algorithm were achieved using 30% of the classes (10 classes) that were identified as most contributing to discriminate between the *SNCA*+/PD+ and control groups. These measures were 65.5% accuracy, 51.7% TPR, 76.9% TNR, and AUC of 0.714. Table 1 shows the ten most contributing classes, ranked in descending order of contribution to the RF classification. The statistical analysis showed that changes in the levels of PC and DG are statistically significant (*p* < 0.05), and GB3 (*p* = 0.0605) and TG (*p* = 0.0588) are nearly statistically significant in differentiating between the two groups. However, correcting for multiple comparisons (testing 34 lipid classes simultaneously) using the Bonferroni adjustment, none of the lipid classes were statistically significant in differentiating between the two groups. The logistic regression revealed that diradylglycerol is significant by the univariate regression (thus, DG appears in bold in Table 1) and glycerophosphatidylcholine and *N*-acylphosphatidylethanolamine are significant by the multivariate regression (thus, PC and NAPE appear in italic in Table 1). Age and sex influence on the classification measures was very low; these variables were ranked 22nd and last of 36 variables, respectively.

**Table 1:** Ten most contributing lipid classes to distinction between *SNCA*+/PD+ and *SNCA*−/PD−.

Rank	Class	Direction	95% CI and *p* value
1	*PC*	Positive	(−17.02, −0.1), *p* value = 0.0475
2	NAPS	Negative	(−69.74, 157.82), *p* value = 0.4395
3	**DG**	Positive	(−16.55, −0.33), *p* value = 0.0418
4	GB3	Negative	(−0.001, 0.045), *p* value = 0.0605
5	Cer	Positive	(−2.37, 0.41), *p* value = 0.1602
6	MhCer	Positive	(−0.73, 0.35), *p* value = 0.4834
7	BMP	Positive	(−0.046, 0.013), *p* value = 0.2592
8	LPS	Positive	(−0.116, 0.041), *p* value = 0.3386
9	TG	Positive	(−19.77, 038), *p* value = 0.0588
10	*NAPE*	Negative	(−0.0004, 0.0032), *p* value = 0.1380

Lipid acronyms: *PC* glycerophosphatidylcholine, *NAPS* *N*-acyl phosphatidylserine, *DG* diradylglycerol, *GB3* globotriaosylceramide, *Cer* ceramide, *MhCer* monohexosylceramide, *BMP* bis(monoacylglycero)phosphate, *LPS* lysophosphatidylserine, *TG* triradylglycerol, *NAPE* *N*-acylphosphatidylethanolamine. Bold or italic font indicate that a class is identified as contributing also by univariate or multivariate regressions, respectively. Directions of impact are based on ridge regression, where positive means that the higher the concentration, the higher the probability of a subject to be classified as *SNCA*+/PD+. 95% CIs and *p* values are reported for the difference between the expected values of the two groups: SNCA+/PD+ and controls.

**Fig. 1:**
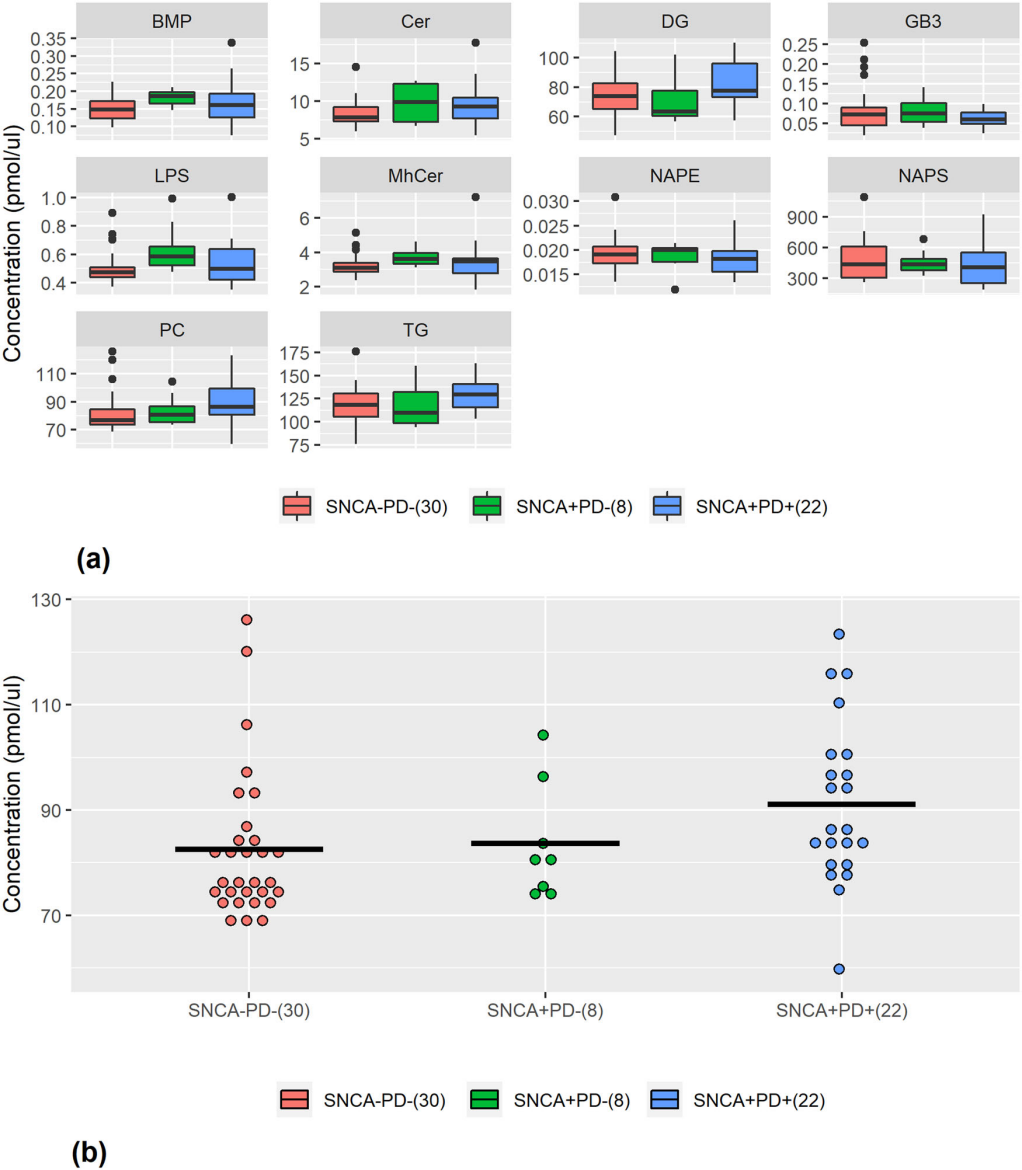
Serum concentrations of the ten most influential lipid classes. Lipid acronyms: PC glycerophosphatidylcholine, NAPS *N*-acyl phosphatidylserine, DG diradylglycerol, GB3 globotriaosylceramide, Cer ceramide, MhCer monohexosylceramide, BMP bis(monoacylglycero)phosphate, LPS lysophosphatidylserine, TG triradylglycerol, NAPE *N*-acylphosphatidylethanolamine. **a** Serum concentrations for the most influential classes as identified by RF (Table 1). The center line represents the median, the bounds of the box are 25th percentile (Q1) and 75th percentile (Q3), the whiskers are Q1 − 1.5*IQR and Q3 + 1.5*IQR, and the dots are outliers. **b** Linear increase in glycerophosphatidylcholine (PC) concentration from *SNCA*−/PD− through *SNCA*+/PD− to *SNCA*+/PD+. Note that subjects of the *SNCA*+/PD− group were not part of the analysis and are shown here only for comparison with the other two groups of subjects. Black lines represent groups’ means. Numbers of observations in parentheses.

Figure 1a presents the serum concentrations of the ten most influential classes (Table 1). The directionality shown in Table 1 and the trends manifested in Fig. 1a are identical. Figure 1b shows concentrations of glycerophosphatidylcholine, which was identified as the most contributing lipid class to discriminate the two groups (ranked first in Table 1). By adding data from *SNCA*+/PD− subjects to the analysis, we demonstrate a linear increase in the glycerophosphatidylcholine concentration from controls through carriers without PD to carriers with PD.

### Lipid *species* in *SNCA*+/PD+

**Fig. 2:**
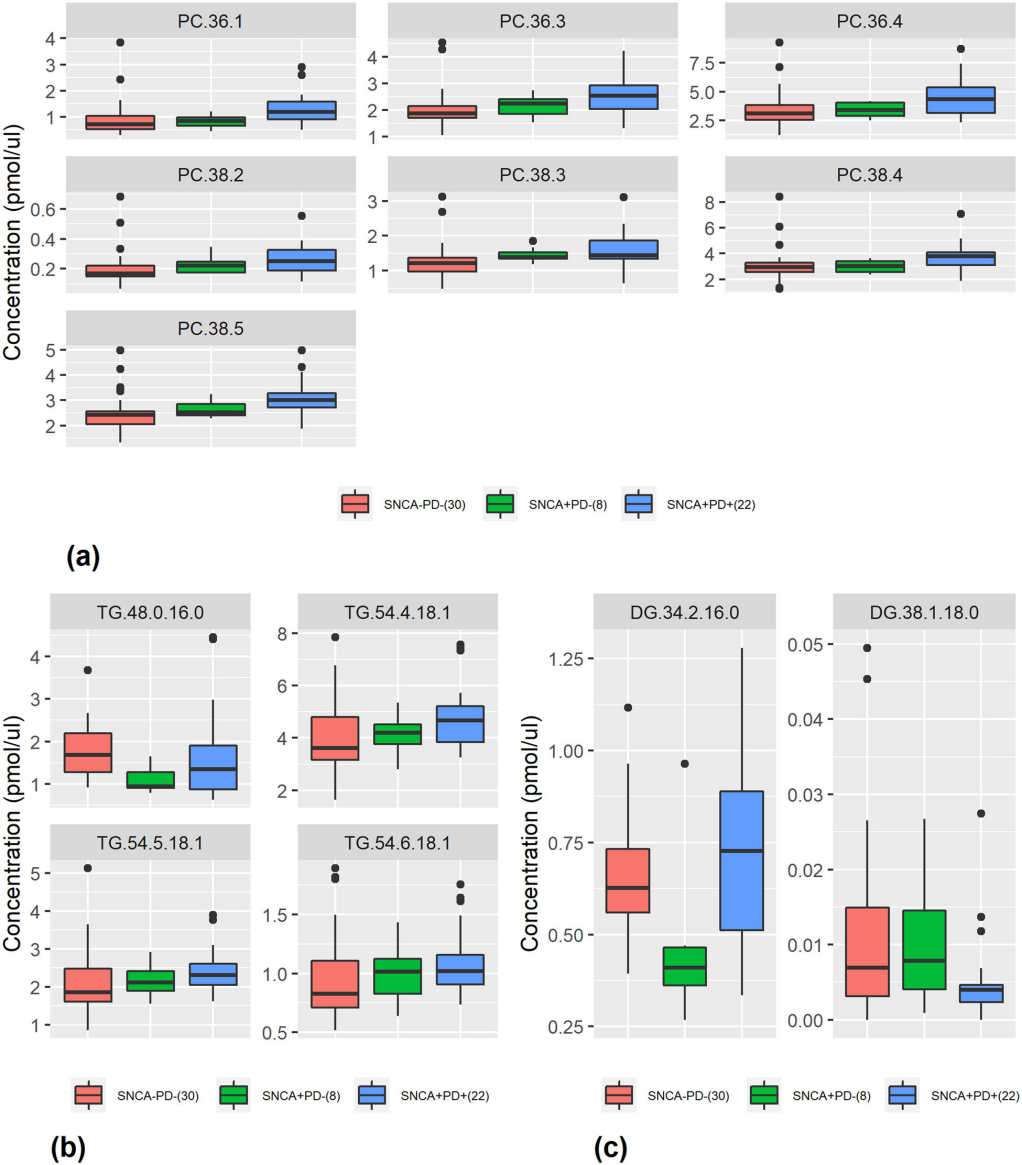
Serum concentrations for contributing glycerophosphatidylcholine, triradylglycerol, and diradylglycerol species. **a**–**c** Concentrations for all contributing glycerophosphatidylcholine (PC), triradylglycerol (TG), and diradylglycerol (DG) species in Table 2. Note that subjects of the *SNCA*+/PD− group were not part of the analysis and are shown here only for comparison with the other two groups of subjects. Note that in all the PC and most of the other species, *SNCA*+/PD− is shown as an intermediate state between the controls and *SNCA*+/PD+ group. The center line represents the median, the bounds of the box are 25th percentile (Q1) and 75th percentile (Q3), the whiskers are Q1 − 1.5*IQR and Q3 + 1.5*IQR, and the dots are outliers.

By selecting the 10 most contributing of the 530 species, together with age and sex (first method in the Material and Methods section), we achieved 78.7% accuracy, 68.5% TPR, 87.3% TNR, and AUC of 0.821. However, by implementing the second method in the Material and Methods section, selecting the 17 most contributing of the 176 species of the 10 most contributing classes (Table 1), we achieved 82.6% accuracy, 73.5% TPR, 89.0% TNR, and AUC of 0.865. Table 2 outlines these 17 species sorted by their contribution to *SNCA*+/PD+ prediction. The first eight species were also identified as influential when all 530 species were considered. Four of them, all from the glycerophosphatidylcholine class, were also identified as significant by the univariate logistic regression. Five of the 17 species have different directions than their classes. Comparison of the average species concentrations between the *SNCA*+/PD+ and control groups by a Welch’s *t* test showed that they were not statistically significant after Bonferroni adjustments (*p* < 0.0029). The majority (~65%) of the contributing species are from two classes: glycerophosphatidylcholine and triradylglycerol. Figure 2a shows clear monotonicity in the increase in the lipid serum concentration for all contributing glycerophosphatidylcholine species (Table 2) when moving from the control group to that of the carriers with PD through the carriers without PD. Similar monotonicity is demonstrated for three of the four contributing triradylglycerol species (Fig. 2b) but not for any of the contributing diradylglycerol species (Fig. 2c).

**Table 2:** Most contributing lipid species of the most contributing classes to distinction between *SNCA*+/PD+ and *SNCA*−/PD−.

Rank	Species	Species direction	Class	Class direction	95% CI and *p* value
1	***PC.38.5***	Positive	PC	Positive	(−0.203, −0.268), *p* = 0.0478
2	***PC.36.3***	Positive	PC	Positive	(−0.961, −0.128), *p* = 0.0116
3	**Cer.d18.1.16.0**	Negative	Cer	Positive	(−0.282, 0.008), *p* = 0.06345
4	**TG.54.5.18.1**	Negative	TG	Positive	(−0.706, 0.101), *p* = 0.1379
5	***PC.36.4***	Positive	PC	Positive	(−1.980, −0.186), *p* = 0.0190
6	**TG.54.6.18.1**	Positive	TG	Positive	(−0.307, 0.064), *p* = 0.1945
7	***PC.38.3***	Positive	PC	Positive	(−0.632, −0.032), *p* = 0.0309
8	**PC.38.4**	Negative	PC	Positive	(−1.364, 0.016), *p* = 0.0552
9	Cer.d18.1.22.0	Positive	Cer	Positive	(−0.330, 0.061), *p* = 0.1733
10	PC.38.2	Positive	PC	Positive	(−0.128, −0.001), *p* = 0.0478
11	*TG.54.4.18.1*	Positive	TG	Positive	(−1.568, −0.087), *p* = 0.0293
12	DG.38.1.18.0	Negative	DG	Positive	(0.0003, 0.011), *p* = 0.0360
13	TG.48.0.16.0	Positive	TG	Positive	(−0.427, 0.619), *p* = 0.7097
14	NAPE.p18.0.22.6.20.4	Negative	NAPE	Negative	(11.e−5,3 .4e−4), *p* = 0.0373
15	PC.36.1	Positive	PC	Positive	(−0.74, 0.00002), *p* = 0.050
16	DG.34.2.16.0	Positive	DG	Positive	(−0.206, 0.569), *p* = 0.2563
17	MhCer.d18.0.26.0	Negative	MhCer	Positive	(−7.5e−6, 6.1e−4), *p* = 0.0556

Lipid acronyms: *PC* glycerophosphatidylcholine, *Cer* ceramide, TG triradylglycerol, *DG* diradylglycerol, *NAPE* *N*-acylphosphatidylethanolamine, *MhCer* monohexosylceramide. Most contributing lipid species of the most contributing classes (Table 1) as ranked by the RF in descending order of influence. Directions of impact are based on ridge regression, where positive means that the higher the concentration, the higher the probability of a subject to be classified as *SNCA*+/PD+. Bold or italic fonts indicate that a species is identified as contributing also when analyzed with all remaining species or by univariate regression, respectively. 95% CIs and *p* values are reported for the difference between the expected values of the two groups: SNCA+/PD+ and controls. The Bonferroni adjustment for multi comparisons is *p* < 0.0029.

To examine whether difference in nutritional status could explain differences in blood lipidome between PD and control samples, we compared total blood cholesterol and phospholipids, two measures that can be used as proxy of lipid nutritional status. As shown in Supplementary Fig. 1, neither significantly differed between controls, *SNCA*+/PD+ and *SNCA*−/PD− samples (controls).

### Lipid changes in *SNCA*^*G209*^-expressing cells and mouse tissues

**Fig. 3:**
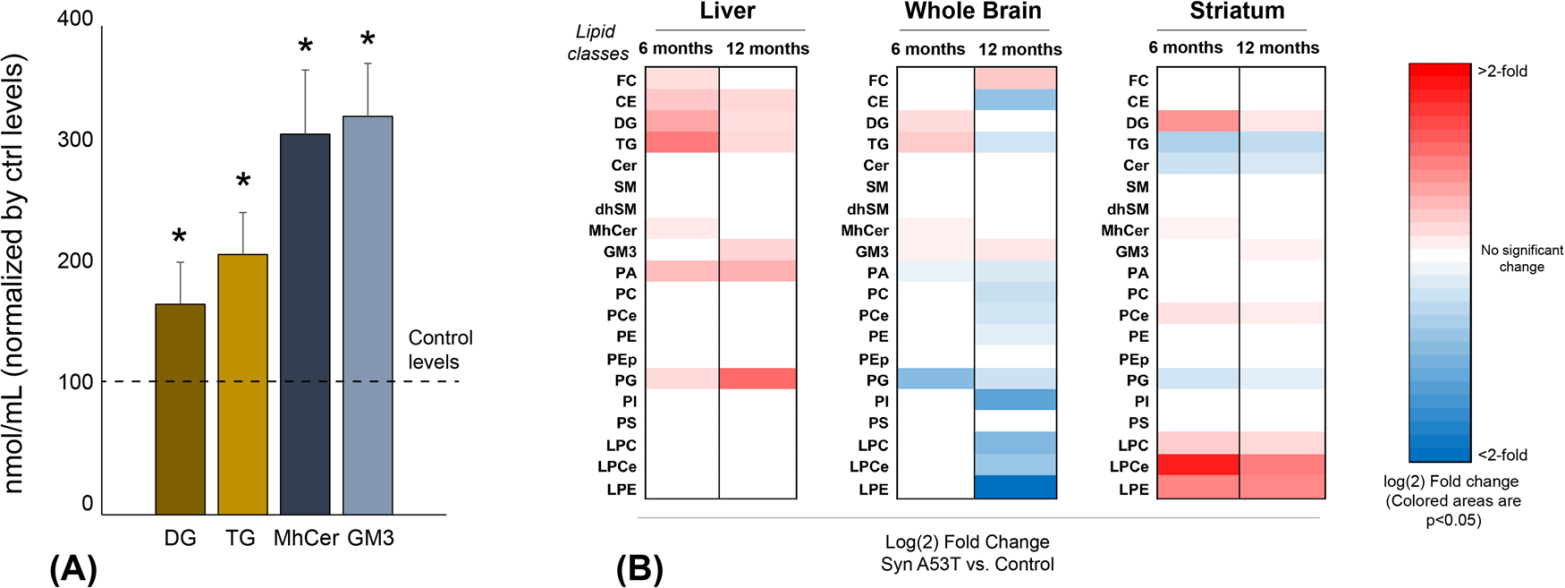
Changes in lipid classes in *SNCA*^*G209A*^ cells and transgenic mice. **a** Elevations in diradylglycerol (DG), triradylglycerol (TG), monohexosylceramide (MhCer), and monosialodihexosylganglioside (GM3) classes in homogenates from *SNCA*^*G209A*^ neuroblastoma cells versus control lines (*n* = 4 biological replicates ± SD each run in triplicates. *p < 0.05, *t* test); **b** heatmaps representing statistically significant changes [log (2) fold change mutant versus controls] in main classes of lipids in tissues from *SNCA*^*G209A*^ transgenic mice versus tissues from *SNCA* transgenic and non-transgenic mice (*n* = 3 biological replicates each run in triplicates. Colored areas are *p* < 0.05. *t* test. CI 95%).

To support the significance of the lipid changes found in PD sera and their link to mutations in *SNCA*, we next analyzed the lipid composition of human neuroblastoma cell lines that stably overexpress either *SNCA*^*G209A*^ or wild-type *SNCA* or endogenous levels of wild-type *SNCA* as well as in transgenic mice overexpressing either *SNCA*^*G209A*^ or wild-type *SNCA* at 6- and 12-months of age as well as in their age-matched non-transgenic littermates. In the mice, lipids were quantified in whole brain and striatum as well as in liver; the latter peripheral organ was included since liver is recognized as playing a critical role in lipid metabolism, hence assessing the liver lipidome provides an excellent proxy of the whole-body lipid homeostasis”^24–26^. With respect to lipid classes, we found significant elevation in diradylglycerol and triradylglycerol levels, as well as in monohexosylceramide and monosialodihexosylganglioside (Fig. 3a), which aligns (for the first three) with the alterations observed in sera of *SNCA*+/PD+ patients. As for our lipidomic analysis in mouse tissues (liver, whole brain, and striatum), we also found (Fig. 3b) increase in diradylglycerol and triradylglycerol levels in liver homogenates of presymptomatic and symptomatic transgenic *SNCA*^*G209A*^ mice and in the two lipid levels in whole brain homogenates of presymptomatic transgenic *SNCA*^*G209A*^ mice. Conversely, homogenates from striatum of presymptomatic and symptomatic transgenic *SNCA*^*G209A*^ mice, while also presenting higher levels of diradylglycerol, show a substantial decrease in triradylglycerol. A decrease in the triradylglycerol levels is also seen in whole brain homogenates of symptomatic transgenic *SNCA*^*G209A*^ mice.

**Fig. 4:**
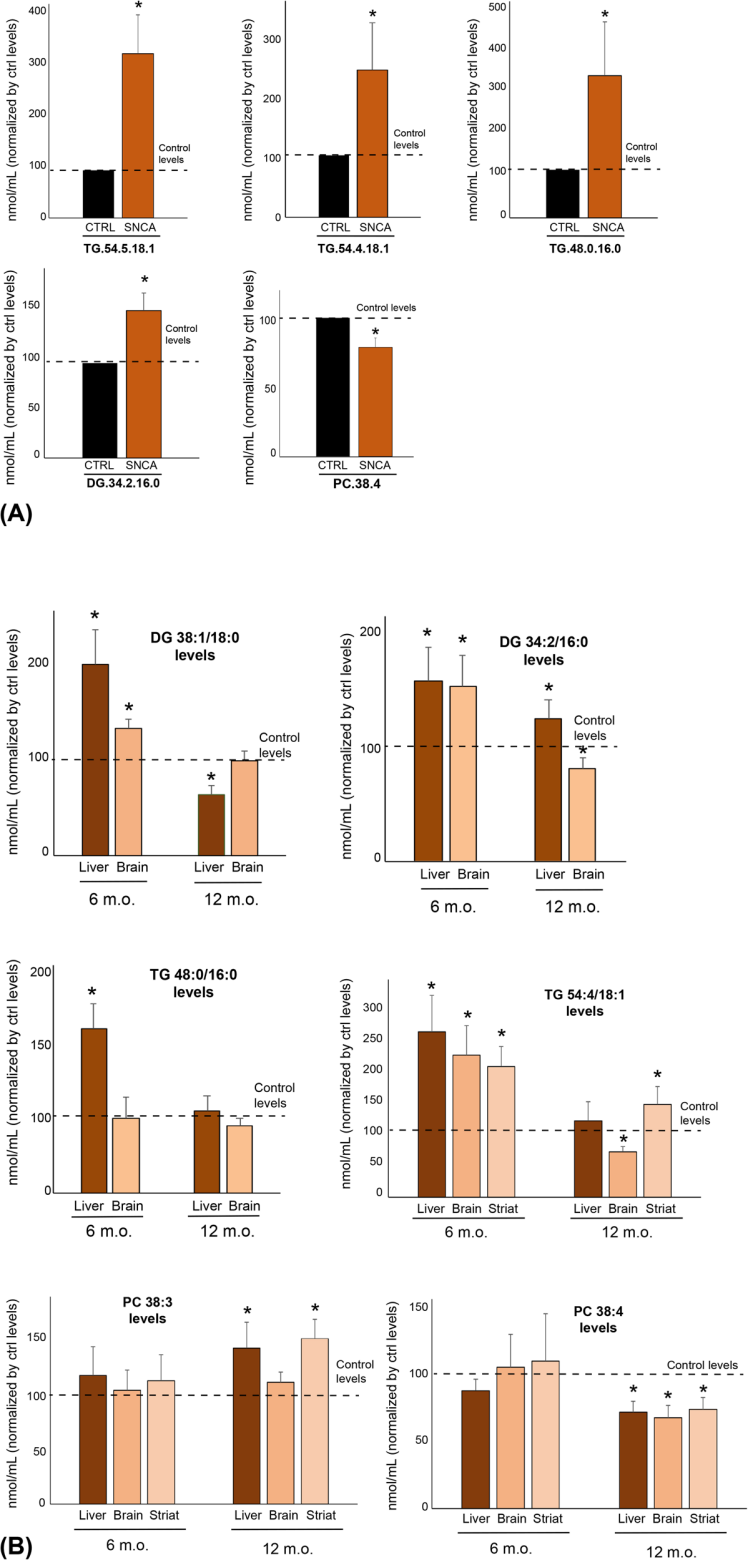
Changes in lipid species in *SNCA*^*G209A*^ cells and transgenic mice: triradylglycerol (TG), glycerophosphatidylcholine (PC), and diradylglycerol (DG). **a** Concentrations of lipid species showing significant changes in *SNCA*^*G209A*^ neuroblastoma cell lines versus control lines (*n* = 4 biological replicates ± SD each run in triplicates. *p < 0.05, *t* test. CI 95%). **b** Concentrations of lipid species in tissues from *SNCA*^*G209A*^ transgenic mice versus tissues from *SNCA* transgenic and non-transgenic mice (*n* = 3 biological replicates ± SD each run in triplicates *p < 0.05. *t* test. CI 95%). The levels of some species were undetectable in striatum samples and were not included in the analysis.

Quantification and analysis of the same 530 lipid species studied in human patients’ serum samples indicate that *SNCA*^*G209A*^ neuroblastoma cells show elevations in triradylglycerol.54.5.18.1, triradylglycerol.54.4.18.1, and triradylglycerol.48.0.16.0, as well as in diradylglycerol.34.2.16.0, while presenting significant decline in the levels of glycerophosphatidylcholine.38.4 (Fig. 4a). Except for the measured levels of triradylglycerol.48.0.16.0 and glycerophosphatidylcholine.38.4, these results support those in human serum samples (Fig. 2). These same triradylglycerol and diradylglycerol species, as well as diradylglycerol.38.1.18:0, were also increased significantly in liver and whole brain tissues of presymptomatic transgenic *SNCA*^*G209A*^ mice (except for triradylglycerol.48.0.16.0 in whole brain) (Fig. 4b). Such increases are also significant for triradylglycerol.54.5.18.1 (liver and striatum), diradylglycerol.34.2.16.0 (liver), and triradylglycerol.54.5.18.1 (liver) in symptomatic transgenic *SNCA*^*G209A*^ mice. Finally, we found an increase in glycerophosphatidylcholine.38.3 in liver and striatum of presymptomatic and symptomatic transgenic *SNCA*^*G209A*^ mice, in agreement with the results obtained in human serum samples (Fig. 2a), and a decrease of glycerophosphatidylcholine.38.4 in all three tissues of these symptomatic animals (and in liver of the presymptomatic mice), which contradicts the results obtained in human serum samples (Fig. 2a).

To determine the effect of overexpression compared to the minor levels of endogenous wild-type aSyn in the parental lines and transgenic animal, we also decided to include in our analysis neuroblastoma cell lines and tissues from animal models overexpressing wild-type α-Syn. Although to a lesser extent, our data indicate that compared to cells and tissues with increased wild-type α-Syn levels (+WT), A53T carriers present with similar lipid alterations to those observed when compared to controls expressing endogenous levels of α-Syn (Supplementary Fig. 2). We note however, that some A53T-associated lipid alterations were also present in cell and tissue models overexpressing wild-type α-Syn. We believe that these changes, rather than a limitation of our study, are the result of the pathogenic role of increased levels of α-Syn.

## Discussion

In the present study, we sought to use ML analysis to identify the lipids that contribute the most to the differentiation between the *SNCA* A53T carriers and control groups. Our analysis showed several influential lipid classes: glycerophosphatidylcholine, triradylglycerol, and diradylglycerol, and, within each class, several lipid species, most of them from the glycerophosphatidylcholine and triradylglycerol classes, that contribute to a more accurate prediction of A53T mutation status.

To strengthen the link between the observed lipid alterations with α-Syn mutation, we extended our lipidomics analysis to neuroblastoma cells lines and tissues from transgenic mice overexpressing the same mutation in α-Syn. While all tissues sampled contain the same genetic mutation, human samples were obtained from the periphery (serum), while cell lines and mouse models aimed to capture central changes in lipid concentrations. This difference in the source of material may account for some of the different outcomes observed. Given the size of the cohort of non-manifesting carriers of the A53T mutation, we cannot be certain whether the changes observed reflect PD status, the mutation, or a combination of the two.

How mutant α-Syn causes lipid alterations remains to be established. Data from our lab has demonstrated that, upon a yet-unknown stimuli, α-Syn can be recruited to certain domains in the ER, called mitochondria-associated ER membranes or MAM^27^. These domains are transient lipid-raft like membranes involved in the regulation of several key cellular functions, including the modulation of multiple lipid enzymes^28–30^. Our previous work has also shown that mutations or overexpression of α-Syn result in its decreased localization to MAM domains and subsequent alterations in the regulation of MAM-resident lipid enzymes^27^. Therefore, it is possible that α-Syn contributes to the regulation of lipid homeostasis via its effect on the modulation of MAM activities. Given that our study focused on A53T models, it remains to be studied if other mutations in *SNCA*, such as copy number variations (duplication and triplications) and other point mutation such as A30P and E46K would lead to a similar lipid profile alteration. With regards to the latter mutation, Rovere and colleagues characterized the molecular pathology of E46K-like α-Syn mutants and demonstrated that curvature selectivity, rather than increased membrane affinity, may be the critical pathology^31^. How curvature selectivity would translate to lipid dysregulation should be studied in future research. Our ML approach identified specific alterations in glycerophosphatidylcholine species, such as elevations in glycerophosphatidylcholine.38.3 and glycerophosphatidylcholine.38.4, as potential identifiers of A53T+ carriers. In our cell and animal model studies, a significant increase in glycerophosphatidylcholine.38,3 was only present in liver and striatum samples from A53T mice, whereas both cell and tissue models exhibited reductions in glycerophosphatidylcholine.38.4. These changes could be the consequence of a change in lipoprotein composition, but they also could imply an alteration in the metabolism of fatty acids. For instance, most glycerophosphatidylcholine.38.3 or glycerophosphatidylcholine.38.4 species are composed of two fatty acids: stearic (C18:0) and mead acid (20:3) or arachidonic acid (20:4), respectively. Therefore, changes in these species could be the consequence of higher 20:3/20:4 ratios. These alterations are caused by deficiencies in essential fatty acids, such as omega-3 polyunsaturated fatty acids^32^. Importantly, low levels of omega-3 affect the brain dopaminergic system^33^, and have been shown to have a neuroprotective effect in the course of PD^34^. Alternatively, decrease in glycerophosphatidylcholine.38.4 could be the result of increased hydrolysis of 20:4 from membrane phospholipids for the activation of inflammatory responses^35^.

*SNCA* A53T+ samples mostly show elevations in diradylglycerol and triradylglycerol concentrations. Similarly, Huang and colleagues^36^ reported that elevated triradylglycerol in patients with PD was associated with mild cognitive impairment, but Fang and colleagues^37^ reported reduced future PD risk in people with higher triradylglycerol levels. Elevated diradylglycerol levels have also been observed in AD patients^38^ suggesting this could be a common feature in different neurodegenerative processes. Triradylglycerol species containing oleic acid (18:1) were particularly elevated in serum samples from *SNCA* A53T+/PD+ patients, as well as in cells and tissues expressing the A53T mutation. Interestingly, these particular species are associated with the activation of *de novo* triradylglycerol synthesis and/or its mobilization from adipose tissues.

Additionally, previous studies have already shown that mutations or overexpression of α-Syn is associated with a significant upregulation of oleic acid-generating enzyme stearoyl-CoA-desaturase (SCD) and the triradylglycerol synthesis enzyme diacylglycerol acyltransferase 2^39^, both of which are modulated at MAM domains in the ER^28–30^. Furthermore, these triradylglycerol species have been associated with decreased insulin sensitivity^40^, which has been previously associated with increased PD risk^41^.

Compared to HDLs, VLDLs and LDLs are enriched in TGs^42^. Thus, our lipidomics data agree with an imbalance of lipoproteins in PD patients. Moreover, our data in mouse tissues suggest that triradylglycerol elevations in blood could be the product of the upregulated formation of VLDL particles as a result of increase in fatty acid synthesis^43^. Alternatively, it is possible that these changes in triradylglycerol levels might be subsequent to a “metabolic reprogramming” that favors glycolytic metabolism over mitochondrial respiration, as observed in other neurodegenerative conditions^44,45^. As such, increase in triradylglycerol would imply a switch towards the use of fatty acids as carbon sources for ATP production. In support of this idea, triradylglycerol elevations, which confer a high risk for cardiovascular disorders, seem to be protective in PD^39^. When sustained, this shift in mitochondrial substrates is quite detrimental for high-energy demanding cells, such as neurons, and induces significant changes in lipid metabolism and membrane composition^46^. Notably, numerous studies have highlighted the role of the lipid composition of cellular membranes in the multimeric conformation and aggregation of α-Syn^47^. Specifically, and in support of our data, α-Syn displays a tendency to associate with lipid membranes enriched in dira- and triradylglycerol^48^ bound to unsaturated fatty acids such as oleic acid^49^.

A major strength of our study is the analysis of a unique cohort of *SNCA* A53T mutation carriers. Analyzing a cohort of carriers of a mutation that predisposes to α-Syn aggregation argues that the lipid alterations may be caused by the alteration in α-Syn metabolism rather than the other way around. However, we did not collect data on potential confounders such as diet or use of statins, which may influence lipid concentrations. As mentioned above, the strength of this unique cohort also holds an inherent sample size limitation. Confirming the findings in larger cohorts of *SNCA* A53T mutation carriers or in cohorts of different mutations in *SNCA* would be imperative given our sample size. To overcome this limitation, another strength of our study is the novel and efficient ML methodology we applied to the data. This methodology offers a solid prediction framework based on the RF classifier by: 1) designing a careful statistically supported training-validation-test ML setting; 2) initially applying the classifier to select from all available lipid classes those contributing to better performance measures and then repeating this application only to those highly ranked classes (and demographics) to select the most contributing lipid species; and 3) validating lipid class and species selection using statistical methods. Lastly, confirming key findings from human samples in cellular and animal models further supports the accuracy of these findings.

A limitation of our study is the lack of information about the nutritional state of the participants, since nutritional status may influence levels of blood lipids^50^. Based on studies on the regulation of lipid metabolism in the cell and the whole body and our own experience^51–53^, the effect of diet on human sera appears negligible compared to the impact of diseases such as PD. Nonetheless, it is relevant to mention the study of Jiang et al., which indicates that less than half of PD patients who are either underweight or overweight have, at worst a mild malnutrition as evidenced by a lower total blood cholesterol levels as compared to controls^54^. Thus, our finding of comparable total blood cholesterol levels between *SNCA*+/PD+ and *SNCA*-/PD- participants (Supplementary Fig. 1A) argues against an overt confounding effect due to nutritional status in the present study. Likewise, we found comparable fasting serum phospholipids among these groups, supporting similar dietary intakes during previous weeks irrespective of recent FA intake^55^ among *SNCA*+/PD+ and *SNCA*−/PD− participants (Supplementary Fig. 1B). Lastly, comparable changes in these lipid species were also observed in A53T+ cells and transgenic mice. Thus, in aggregate we believe that our data provide compelling evidence that mutant α-Syn is associated with alterations in lipidome.

Our study, however, cannot infer causality. We cannot determine if the observed lipid alterations contribute to the pathogenesis of PD. Specifically, the non-manifesting carrier group (*SNCA*+/PD−) was too small to be included in detailed analyses. Therefore, it is hard to separate the mutation effect from PD status effect. Future studies should further investigate whether alterations of glycerophosphatidylcholine, diradylglycerol, and triradylglycerol may contribute to enhanced α-Syn aggregation. Specifically, future studies should test if modifying the concentrations of these lipids “to normalize” these to the concentration of non-carriers might reduce α-Syn aggregation and the progression of PD.

## Methods

### Participants

Subjects harboring the G209A/p.A53T mutation in the *SNCA* gene (designated as *SNCA*+/PD+ hereafter, *n* = 30) as well as age and sex-matched healthy controls (*n* = 30), were separately recruited in the MEFOPA study (Mendelian Forms Of Parkinsonism)^56^. Subjects with the mutation were classified as symptomatic (*SNCA*+/PD+, *n* = 22) or asymptomatic carriers (*SNCA*+/PD‒, *n* = 8). The analysis was conducted on 52 subjects: 22 PD patients (*SNCA*+/PD+) and 30 healthy controls (*SNCA*‒/PD‒). The sex, age, age at on set, and other PD patients’ characteristics are presented in Table 3. The two groups are not significantly different with respect to sex (*p* = 0.549) and age (*p* = 0.711). The eight *SNCA*+/PD‒ subjects were only included in a secondary analysis aimed at examining the potential effect of *SNCA* mutations on the lipidome, irrespective of the PD phenotype.

**Table 3:** Demographic and Parkinson’s disease characteristics of the cohort.

Characteristics	*SNCA*+/PD+ (*n* = 22)		*SNCA*−/PD− (*n* = 30)		*SNCA*+/PD− (*n* = 8)	
Sex	55% female		67% female		88% female	
	**Mean (Std)**	**Median (Min, Max)**	**Mean (Std)**	**Median (Min, Max)**	**Mean (Std)**	**Median (Min, Max)**
Age (years)	50.8 (10.8)	51 (32, 67)	52.1 (13.4)	52.5 (31, 81)	50.1 (18.5)	43 (34, 87)
Age at onset (years)	44.8 (10.3)	45 (30, 65)				
PD duration (years)	6.2 (4.5)	5 (3, 16)				
H&Y		2 (1, 5)				
UPDRS III	30.9 (28.1)	20 (4, 91)				
MocA	25.8 (3.5)	26.5 (18, 31)				

*H&Y* Hoehn and Yahr, *UPDRS* Unified Disease Rating Scale; MocA: Montreal Cognitive Assessment.

### Clinical assessment

The clinical assessment of the *SNCA* carriers at baseline and the time of the blood draw have been described in detail by Papadimitriou and colleagues^57^. This includes all carriers, symptomatic and asymptomatic.

### Ethics statement

All study procedures were approved by the scientific council and ethical committee of Attikon Hospital and all participants provided written informed consent.

### Collection of serum

Blood from human subjects was collected in clot activator-coated tubes (BD Vacutainer, Ref #367986). Each sample was centrifuged at 2200 x g for 10 min, and the supernatant was collected. Serum was aliquoted in 2 mL polypropylene tubes and stored at -80^o^C until used.

### Detection of *SNCA*^*G209A*^ in blood cells

DNA was extracted from peripheral blood and was analyzed for the presence of the *SNCA* A53T mutation, as well as for additional *SNCA*, *LRRK2*, *PRKN*, *PINK-1* and *DJ-1* mutations by Sanger sequencing^56^.

### Lipid analysis

For lipidomic analysis, we extracted lipids from serum aliquots that had not been previously thawed. Lipids were extracted from equal amounts of material (0.2 mL/sample) and subjected to a chloroform–methanol extraction by modified Bligh and Dyer protocol^52,58^. Three different aliquots from each sample were analyzed in singleton by Liquid Chromatography coupled to Mass Spectrometry as previously described^52^. After identification and alignment of the detected lipid peaks, which consistently corresponded to 530 different species from 34 different classes (Supplementary Table 1), each individual peak was normalized by applying the Normalization using Optimal selection of Multiple Internal Standard method or NOMIS (Supplementary Table 2)^59^. The normalized data sets were then subjected to the RF classifier to identify and rank lipid classes and species that differentiated most between the *SNCA*+/PD+ and *SNCA*-/PD- groups. The RF classifier holds no assumptions about the data distribution and can cope with very complex problems with minimum overfitting^14,15,60^, and ranks variables by their contribution to accurate (or informative) prediction^61–63^. In the lipid species analysis, we implemented two different methods. In the first method, we chose the most contributing species from all species, and in the second method, in the intent of improving the classification results, we exploited the classes analysis results by using only the species derived from the most contributing classes (Table 1). Next, we validated these results using several well-known statistical methods including univariate and multivariate logistic regressions, implemented by considering variables using sequential backward selection, sequential forward selection, or sequential floating forward selection, and Ridge regression^64^. Last, for the identified classes and species, we compared distributions of serum concentration between *SNCA*+/PD+ and *SNCA*−/PD− using a two-tailed Welch’s *t* test assuming non-equal variances and a 95% confidence level (CI). Note that our results are adjusted for multiple comparisons for the RF by the Monte Carlo cross validation and randomization of the tests methodologies (i.e., data sampling in creating each tree of the RF), and for the Welch’s *t* test by the Bonferroni correction^65^. The performance measures that include accuracy, true positive rate (TPR) (sensitivity), true negative rate (TNR) (specificity), and area under the curve (AUC) were calculated using Monte Carlo cross validation of 200 datasets. In each dataset, 80% of the observations were sampled randomly (without replacement) for training the algorithm, and the remaining 20% were used for testing it. Optimization of the RF algorithm (i.e., tuning its hyper-parameters) was done using a validation set formed by splitting the training set randomly to actual training and validation sets in an 80:20% ratio. This resampling method was chosen to reinforce the significance of the results when experimenting with the small cohort. The data that support the findings of this study are available from the corresponding author upon request.

### Analysis of cells and mouse lines

To validate the association between *SNCA*+/PD+ status and the changes in serum lipidome, we used both cell lines and transgenic mouse model of *SNCA*^*G209A*^. Cells used in this work are human BE(2)-M17 neuroblastoma, which stably overexpress either mutant *SNCA*^*G209A*^ or wild-type *SNCA* or express endogenous levels of wild-type *SNCA* (kindly provided by Erwan Bezard, Université de Bordeaux). The characteristics of these two cell lines were detailed by Bisaglia et al (2010)^66^. We also used transgenic mice overexpressing either mutant *SNCA*^*G209A*^ (B6; C3-Tg (Prnp-SNCA*A53T)83Vle/J, The Jackson Laboratory, Bar Harbor, ME) or wild-type *SNCA* (Line M7; stock no. 010710; FVB The Jackson Laboratory, Bar Harbor, ME) as well as their non-transgenic littermates. Mice (*n* = 3 per group) were males and females of 6 and 12 months of age at the time of analysis; these two time points were selected as they reflect the pre-symptomatic and symptomatic stages of the disease phenotype in these mouse lines. The characteristics of these transgenic mice were previously described by Giasson et al.^67^.

### Reporting summary

Further information on research design is available in the Nature Research Reporting Summary linked to this article.

## Supplementary information

Supplementary material

Reporting Summary

## Data Availability

The datasets generated during and/or analyzed during the current study are available from the corresponding author on reasonable request.
