# Supplementary material for: Lipid level alteration in human and cellular models of alpha synuclein mutations
Source: NPJ Parkinsons Dis. 2022 Apr 25;8:52. doi: 10.1038/s41531-022-00313-y (PMC9039073; doi:10.1038/s41531-022-00313-y)
Supplement: Supplementary file 1 — Supplementary material [file 41531_2022_313_MOESM1_ESM.pdf]

Lipid Level Alteration in Human and Cellular Models of Alpha Synuclein Mutations

Hila Avisar[1*], Cristina Guardia-Laguarta[2*], Matthew Surface[2], Nikos Papagiannakis[3,4], Matina Maniati[4], Roubina Antonellou[3,5], Dimitra Papadimitriou[6], Christos Koros[3,5], Aglaia Athanassiadou[7], Serge Przedborski[2,8], Boaz Lerner[1], Leonidas Stefanis[3,4,5], Estela Area-Gomez[2], Roy N. Alcalay[2, 9]

Supp. Table 1: Lipids measured in the study.

Type	Abbrev.	Full name
Neutral Lipids	FC	Cholesterol
	CE	Cholesterol Ester
	AC	Acyl Carnitine
	MG	Monoradylglycerol
	DG	Diradylglycerol
	TG	Triradylglycerol
Sphingolipids	Cer	Ceramide
	dhCer	Dihydroceramide
	SM	Sphingomyelin
	dhSM	Dihydrosphingomyelin
	Sulf	Sulfatide
	MhCer	Monohexosylceramide
	LacCer	Lactosylceramide
	GM3	Monosialodihexosylganglioside
	GB3	Globotriaosylceramide
Phospholipids	PA	Glycerophosphatidic acid
	PC	Glycerophosphatidylcholine
	PCe	Ether Glycerophosphatidylcholine
	PE	Phosphatidylethanolamine
	PEp	Plasmalogen Glycero phosphatidylethanolamine
	PG	Glycerophosphatidylglycerol
	PI	Glycerophosphatidylinositol
	PS	Glycerophosphatidylserine
	LPC	Lysophosphatidylcholine
	LPCe	Ether Lysophosphatidylcholine
	LPE	Lysophosphatidylethanolamine
	LPEp	Plasmalogen Lysophosphatidylethanolamine
Phospholipids	LPI	Lysophosphatidylinositol
	LPS	Lysophosphatidylserine
	BMP	Bis(monoacylglycero)phosphate
	AcylPG	Acyl Phosphatidylglycerol
	NAPE	N-Acylphosphatidylethanolamine
	NSer	N-Acyl Serine
	NAPS	N-Acyl Phosphatidylserine

Supp. Table 2: Lipid internal standards used in this lipidomics study

Internal Standard	Corresponding Lipid Class	Concentration (ug/ul)
IS AcylPG 14:0-28:0	Acyl PG, NAPE, NAPS	0.046799614
IS BMP 28:0	BMP	0.015298133
IS CE C17	CE	78.59098931
IS Cer C17:0	Cer, dhCer	0.758320608
IS Chol d7 b	Free Cholesterol	63.78791732
IS DG 4ME	diacylglycerols	0.640874053
IS dhSM d18:0/12:0	dihydrosphingomyelins	2.579623778
IS DMPC	AC	12.34642208
IS GalCer d18:1/12:0	MhCer	1.039897431
IS LacCer d18:1/12:0	LacCer	0.259594347
IS LPC 13:0	LPC	12.34642208
IS LPE 14:0	LPE	0.098349468
IS LPI 13:0	LPI	0.07642123
IS MG C17	MG	0.242952978
IS PA 28:0	PA	0.068072997
IS PC 28:0	PC	12.34642208
IS PE 25:0	PE	8.839285714
IS PG 12:0/13:0	PG	0.446428571
IS PI 12:0/13:0	PI	2.232142857
IS PS 28:0	PS	11.92531331
IS SM d18:1/12:0	SM	13.39285714
IS Sulf d18:1/12:0	Sulf	0.225924621
IS TG 50:0 d5	TG	0.498018035

Supp. Figure 1. Comparison of the concentrations of Cholesterol and Cholesterol Ester between SNCA-/PD- and SNCA-/PD-

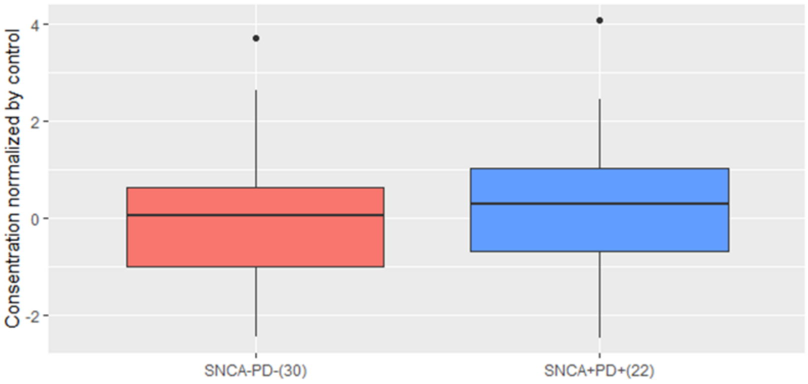

(A)

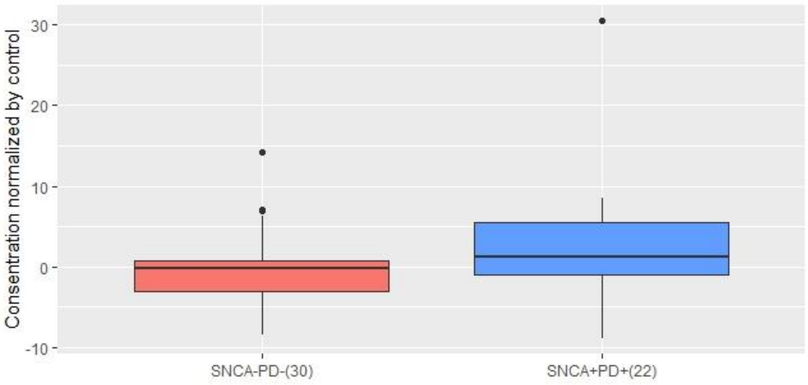

(B)

(A). Sum of plasma concentrations of Cholesterol (FC) and Cholesterol Ester (CE) each is first z-score normalized according to the SNCA-/PD- group provided for both groups. The difference between the groups is insignificant (p=0.5745). The center line represents the median, the bounds of the box are 25th percentile (Q1) and 75th percentile (Q3), the whiskers are Q1-1.5*IQR and Q3+1.5*IQR, and the dots are outliers.

center line, bounds of box and whiskers

(B). The same for the Phospholipids: Glycerophosphatidic acid (PA), Glycerophosphatidylcholine (PC), Ether Glycerophosphatidylcholine (PCe), Phosphatidylethanolamine (PE), Plasmalogen Glycero phosphatidylethanolamine (PEp), Glycerophosphatidylglycerol (PG), Glycerophosphatidylinositol (PI), and Glycerophosphatidylserine (PS). The difference between the groups is insignificant (p=0.1194).

Supp. Figure 2

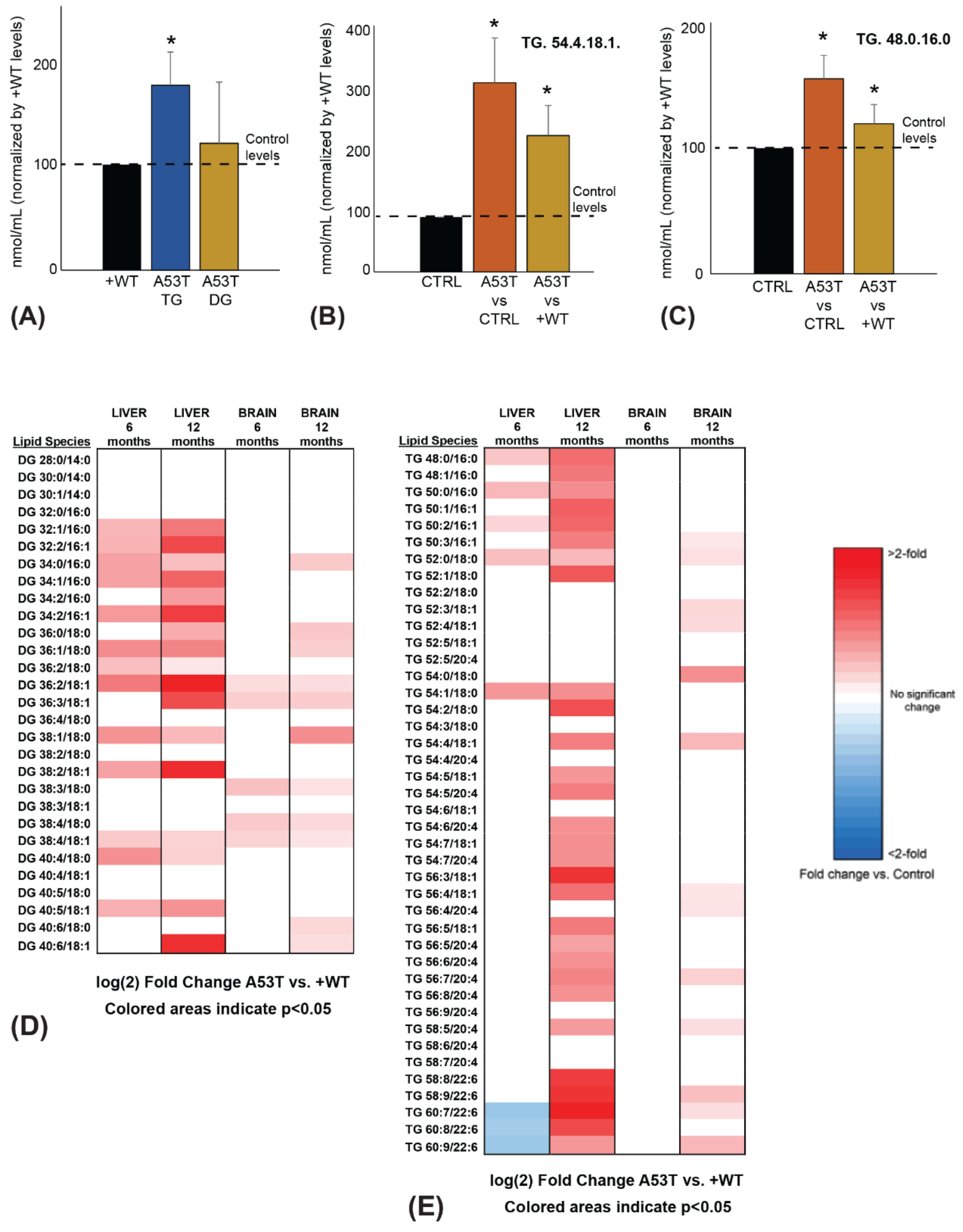

Supp. Fig. 2. (**A**) Comparison of total TG and DG levels between cells overexpressing Syn-A53T or Syn-WT. (**B, C**) Analysis of the levels of the indicated species in cells expressing endogenous levels of Syn (CTRL) or overexpressing WT-Syn (+WT); (**D, E**) Heatmaps representing statistically significant changes [log (2) fold change mutant versus controls] in DG (D) and (TG)

species in tissues from *SNCA*^{G209A} transgenic mice versus tissues from *SNCA*^{WT} transgenic (n=3 biological replicates each run in triplicates. Colored areas are $p<0.05$. t-test. CI 95%)
