# Supplementary material for: Lipid level alteration in human and cellular models of alpha synuclein mutations
Source: NPJ Parkinsons Dis. 2022 Apr 25;8:52. doi: 10.1038/s41531-022-00313-y (PMC9039073; doi:10.1038/s41531-022-00313-y)
Supplement: Supplementary file 2 — Reporting Summary [file 41531_2022_313_MOESM2_ESM.pdf]

Corresponding author(s): ROY ALCALAY

Last updated by author(s): Feb 15, 2022

Reporting Summary

Nature Portfolio wishes to improve the reproducibility of the work that we publish. This form provides structure for consistency and transparency in reporting. For further information on Nature Portfolio policies, see our Editorial Policies and the Editorial Policy Checklist.

Statistics

For all statistical analyses, confirm that the following items are present in the figure legend, table legend, main text, or Methods section.

n/a	Confirmed	
☐	☒	The exact sample size (*n*) for each experimental group/condition, given as a discrete number and unit of measurement
☐	☒	A statement on whether measurements were taken from distinct samples or whether the same sample was measured repeatedly
☐	☒	The statistical test(s) used AND whether they are one- or two-sided<br>*Only common tests should be described solely by name; describe more complex techniques in the Methods section.*
☐	☒	A description of all covariates tested
☐	☒	A description of any assumptions or corrections, such as tests of normality and adjustment for multiple comparisons
☐	☒	A full description of the statistical parameters including central tendency (e.g. means) or other basic estimates (e.g. regression coefficient) AND variation (e.g. standard deviation) or associated estimates of uncertainty (e.g. confidence intervals)
☐	☒	For null hypothesis testing, the test statistic (e.g. *F*, *t*, *r*) with confidence intervals, effect sizes, degrees of freedom and *P* value noted<br>*Give P values as exact values whenever suitable.*
☒	☐	For Bayesian analysis, information on the choice of priors and Markov chain Monte Carlo settings
☐	☒	For hierarchical and complex designs, identification of the appropriate level for tests and full reporting of outcomes
☒	☐	Estimates of effect sizes (e.g. Cohen's *d*, Pearson's *r*), indicating how they were calculated

Our web collection on statistics for biologists contains articles on many of the points above.

Software and code

Policy information about availability of computer code

Data collection	*Provide a description of all commercial, open source and custom code used to collect the data in this study, specifying the version used OR state that no software was used.*
Data analysis	*Provide a description of all commercial, open source and custom code used to analyse the data in this study, specifying the version used OR state that no software was used.*

For manuscripts utilizing custom algorithms or software that are central to the research but not yet described in published literature, software must be made available to editors and reviewers. We strongly encourage code deposition in a community repository (e.g. GitHub). See the Nature Portfolio guidelines for submitting code & software for further information.

Data

Policy information about availability of data

All manuscripts must include a data availability statement. This statement should provide the following information, where applicable:

- Accession codes, unique identifiers, or web links for publicly available datasets
- A description of any restrictions on data availability
- For clinical datasets or third party data, please ensure that the statement adheres to our policy

The data that support the findings of this study are available from the corresponding author upon request.

Field-specific reporting

Please select the one below that is the best fit for your research. If you are not sure, read the appropriate sections before making your selection.

☒ Life sciences ☐ Behavioural & social sciences ☐ Ecological, evolutionary & environmental sciences

For a reference copy of the document with all sections, see nature.com/documents/nr-reporting-summary-flat.pdf

Life sciences study design

All studies must disclose on these points even when the disclosure is negative.

Sample size	Sample size was a result of limited samples. Clearly sateted
Data exclusions	No data was excluded
Replication	replication took place in the animal models as stated
Randomization	n/a
Blinding	lipidomic lab was blinded to Parkinson's status of samples

Reporting for specific materials, systems and methods

We require information from authors about some types of materials, experimental systems and methods used in many studies. Here, indicate whether each material, system or method listed is relevant to your study. If you are not sure if a list item applies to your research, read the appropriate section before selecting a response.

Materials & experimental systems

n/a	Involved in the study	
☒	☐	Antibodies
☐	☒	Eukaryotic cell lines
☒	☐	Palaeontology and archaeology
☐	☒	Animals and other organisms
☐	☒	Human research participants
☒	☐	Clinical data
☒	☐	Dual use research of concern

Methods

n/a	Involved in the study	
☒	☐	ChIP-seq
☒	☐	Flow cytometry
☒	☐	MRI-based neuroimaging

Eukaryotic cell lines

Policy information about cell lines

Cell line source(s)	Cells used in this work are human BE(2)-M17 neuroblastoma, which stably overexpress either mutant SNCAG209A or wild-type SNCA or express endogenous levels of wild-type SNCA (kindly provided by Erwan Bezard, Université de Bordeaux). The characteristics of these two cell lines were detailed by Bisaglia et al (2010).
Authentication	Cells used in this work are human BE(2)-M17 neuroblastoma, which stably overexpress either mutant SNCAG209A or wild-type SNCA or express endogenous levels of wild-type SNCA (kindly provided by Erwan Bezard, Université de Bordeaux). The characteristics of these two cell lines were detailed by Bisaglia et al (2010).
Mycoplasma contamination	cell lines were negative to mycoplasma
Commonly misidentified lines (See ICLAC register)	n/a

Animals and other organisms

Policy information about studies involving animals; ARRIVE guidelines recommended for reporting animal research

Laboratory animals	We also used transgenic mice overexpressing either mutant SNCAG209A (B6; C3-Tg (Prnp-SNCA*A53T)83Vle/J, The Jackson Laboratory, Bar Harbor, ME) or wild-type SNCA (Line M7; stock no. 010710; FVB The Jackson Laboratory, Bar Harbor, ME) as well as their non-transgenic littermates.
Wild animals	n/a

Field-collected samples	*For laboratory work with field-collected samples, describe all relevant parameters such as housing, maintenance, temperature, photoperiod and end-of-experiment protocol OR state that the study did not involve samples collected from the field.*
Ethics oversight	All procedures were performed according to the animal study protocols approved by the Institutional Animal Care and Use Committee of the Columbia University in the city of New York, USA.

Note that full information on the approval of the study protocol must also be provided in the manuscript.

Human research participants

Policy information about studies involving human research participants

Population characteristics	Subjects harboring the G209A/p.A53T mutation in the SNCA gene (designated as SNCA+/PD+ hereafter, n=30) as well as age and sex-matched healthy controls (n=30), were separately recruited in the MEFOPA study (Mendelian Forms Of Parkinsonism).25 Subjects with the mutation were classified as symptomatic (SNCA+/PD+, n=22) or asymptomatic carriers (SNCA+/PD–, n=8). The analysis was conducted on 52 subjects: 22 PD patients (SNCA+/PD+) and 30 healthy controls (SNCA–/PD–).
Recruitment	Subjects harboring the G209A/p.A53T mutation in the SNCA gene (designated as SNCA+/PD+ hereafter, n=30) as well as age and sex-matched healthy controls (n=30), were separately recruited in the MEFOPA study (Mendelian Forms Of Parkinsonism).25 Subjects with the mutation were classified as symptomatic (SNCA+/PD+, n=22) or asymptomatic carriers (SNCA+/PD–, n=8). The analysis was conducted on 52 subjects: 22 PD patients (SNCA+/PD+) and 30 healthy controls (SNCA–/PD–).
Ethics oversight	All study procedures were approved by the scientific council and ethical committee of Attikon Hospital and all participants provided written informed consent.

Note that full information on the approval of the study protocol must also be provided in the manuscript.
